# A new spray-type adhesion barrier (AdSpray) improves condition for surgical treatment in the reversal of Hartmann procedure: A case report

**DOI:** 10.1097/MD.0000000000028000

**Published:** 2021-12-03

**Authors:** Wataru Osumi, Masashi Yamamoto, Shinsuke Masubuchi, Hiroki Hamamoto, Masatsugu Ishii, Keisuke Izuhara, Kohei Taniguchi, Toru Kuramoto, Yusuke Suzuki, Keitaro Tanaka, Junji Okuda, Kazuhisa Uchiyama

**Affiliations:** a Department of General and Gastroenterological Surgery, Osaka Medical and Pharmaceutical University, 2-7 Daigaku-machi, Takatsuki, Osaka, Japan; b Translational Research Program, Osaka Medical and Pharmaceutical University, 2-7 Daigaku-machi, Takatsuki, Osaka, Japan; c Cancer Center, Osaka Medical and Pharmaceutical University, 2-7 Daigaku-machi, Takatsuki, Osaka, Japan.

**Keywords:** Adspray, case report, reversal of Hartmann procedure, single incision laparoscopic surgery

## Abstract

**Rationale:**

Hartmann procedure (HP) often causes severe postoperative adhesions in the pelvic space; therefore, the reversal of Hartmann procedure (RHP) is a challenging surgery. A new spray-type antiadhesion agent, AdSpray, has been reported to be useful in three-dimensional fields such as the liver. However, there are no reports of its use in HP. We present a case of a male patient with rectal cancer who underwent laparoscopic HP with AdSpray to prevent postoperative adhesions.

**Patient concerns:**

A 52-year-old man presented with melena and constipation.

**Diagnosis:**

Colonoscopy revealed an almost obstructive type II tumor at the rectosigmoid colon, and histopathological examination revealed moderately differentiated adenocarcinoma. Enhanced abdominal computed tomography revealed slightly enlarged regional lymph nodes but no ascites around the tumor, and there was no metastasis to the liver or lungs. Therefore, we diagnosed clinical stage T4aN1bM0 rectosigmoid colon cancer. Intraoperatively, a metastatic tumor of the liver surface and a high degree of valve retention in the oral colon were identified.

**Interventions:**

After performing laparoscopic HP with AdSpray, we scheduled a laparoscopic RHP with staged hepatic surgery for synchronous liver metastasis from colorectal cancer 1 month later.

**Outcomes:**

No postoperative inflammatory adhesions were observed in the pelvis or around the rectal stump, allowing us to perform RHP by a single-incision laparoscopic surgery from the stoma site without any problem. The operation time for RHP was 80 minutes; the patient was in good general condition after the operation, and he was discharged on postoperative day 7.

**Lessons:**

In laparoscopic HP, Adspray was easy to use for three-dimensional fields such as the pelvis and effectively prevented postoperative inflammatory adhesions. Thus, RHP may become less risky and be performed more as a minimally invasive surgery.

## 1 Introduction

Hartmann procedure (HP) involves resection of the rectosigmoid colon, followed by the closure of the rectal segment and the creation of an end colostomy. In 1921, Henri Hartmann^[1]^ detailed the procedure for left-sided obstructive colorectal cancer to avoid a high degree of anastomotic leakage and mortality and it is now widely used for emergency colonic diseases such as obstructive left-sided colorectal cancer involving perforation and peritonitis, diverticulitis, and ischemia. However, reversal of Hartmann procedure (RHP), which restores intestinal continuity, is associated with severe peri- and postoperative complications, mainly due to a high degree of postoperative adhesions in the initial surgery.^[2]^ Therefore, there are many cases of permanent colostomy without RHP.

In recent years, various drugs have been developed to prevent postoperative adhesions, and there have been many reports on their effective use.^[3,4]^ However, the pelvis is a three-dimensional field and most of the existing sheet-type antiadhesion agents do not provide a clean coating. A new type of antiadhesion agent spray, AdSpray (Terumo, Tokyo, Japan), is reportedly useful in other three-dimensional fields such as the liver region.^[5]^ However, there are no reports of its use in HP.

Herein, we report on the case of a male patient who underwent treatment with AdSpray to prevent postoperative adhesions after HP. No pelvic adhesions were observed at the RHP performed 1 month later and the patient underwent single-incision laparoscopic surgery (SILS) from the stoma site without any complications. Therefore, we consider the use of AdSpray useful in preventing pelvic adhesions, which are the main drawback when considering RHP.

## 2 Case presentation

A 52-year-old man presented with melena and constipation and underwent colonoscopy. An almost obstructive type II tumor was found at the rectosigmoid. Histopathological examination revealed moderately differentiated adenocarcinoma. Therefore, the patient was referred to our department for surgical treatment.

The patient had been receiving treatment for diabetes mellitus and asthma since 10 years. A physical examination revealed abdominal distension and slight left lower abdominal tenderness. He had a normal temperature, with otherwise normal vital signs. Blood test results revealed that his carcinoembryonic antigen level was 19.0 mg/dL, but all other blood test results were within the normal range. Enhanced abdominal computed tomography revealed a high-density area in the rectosigmoid colon. There were slightly enlarged regional lymph nodes but no ascites around the tumor, and there was no metastasis to the liver or lungs. Based on these findings, the patient was diagnosed with clinical stage T4aN1bM0 rectosigmoid colon cancer. For treatment, we proposed laparoscopic anterior resection with regional lymph node resection.

**Figure 1:**
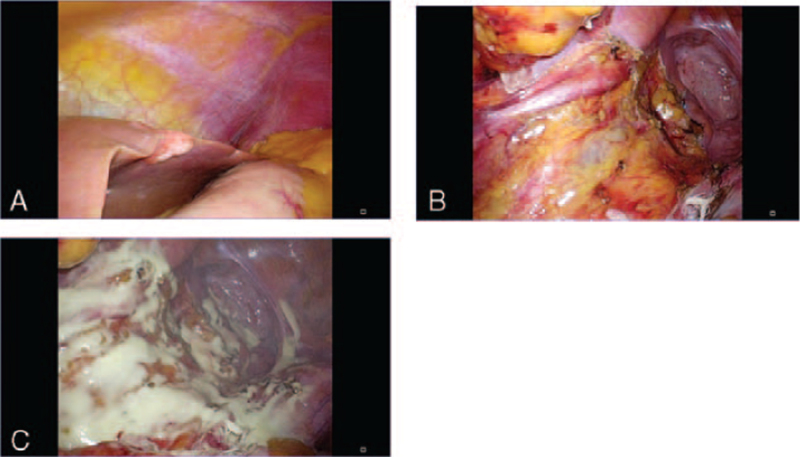
Representative intraoperative laparoscopic images during Hartmann procedure. (A) Intraoperative image showing a metastatic tumor at the surface of the lateral segment of the liver. (B) Intraoperative images showing the pelvic floor and the regional lymph node dissection area before and (C) after using Adspray.

Intraoperatively, a metastatic tumor on the surface of the lateral segment of the liver (Fig. 1A) and a high degree of valve retention in the oral colon were identified. A staged hepatic surgery for synchronous liver metastasis from colorectal cancer was planned and we performed HP for the primary tumor and the regional lymph node (Fig. 1B). We decided to use Adspray to prevent postoperative pelvic adhesions, and Adspray was prepared within 1 hour before use. The planned areas to be sprayed were the pelvic cavity and dissection area of regional lymph node. First, planned site was sufficiently irrigated with warm saline to remove clots. Then, sufficient suction was applied, the water was thoroughly wiped off with laparoscopic gauze, and Adspray was sprayed into the pelvic cavity and the dissected area of the regional lymph node (Fig. 1C). The surgery was completed, and the operative time was 165 minutes with minimal blood loss.

The patient's postoperative course was uncomplicated and he was discharged from the hospital on postoperative day 10. After discharge, magnetic resonance imaging was performed. It revealed no radiological evidence of another metastatic liver tumor. He strongly requested that the colostomy be closed as soon as possible; therefore, RHP and laparoscopic partial resection of the liver were conducted simultaneously 1 month later.

**Figure 2:**
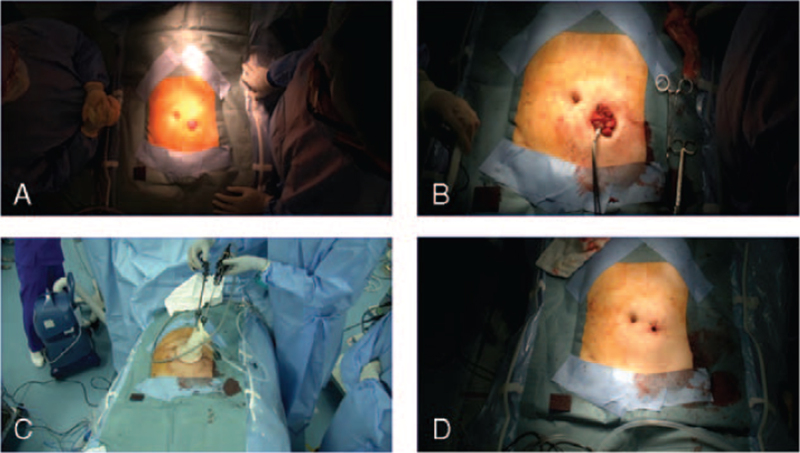
Representative intraoperative images during the reversal of Hartmann procedure. (A) Intraoperative image showing the start of the reversal of Hartmann procedure and (B) the end of the procedure. (C) The anvil of EEA 28 mm is fixed and (D) single-incision laparoscopic surgery is performed from the stoma site.

Due to the risk of postoperative infection, we decided to perform the 2 surgeries in separate settings, and the RHP was performed first. After the induction of anesthesia, the patient was placed in the lithotomy position (Fig. 2A). First, the endocolostomy was closed and prepared circularly in the subcutaneous tissue and fascial layer for mobilization. A purse string clamp was placed 3 cm under the end of the bowel, and the anvil of Tri-Staple EEA circular 28 mm (Johnson & Johnson, New Brunswick, NJ) was fixed by closing the purse string suture (Fig. 2B). Then the end of the oral side was returned to the abdominal cavity.

**Figure 3:**
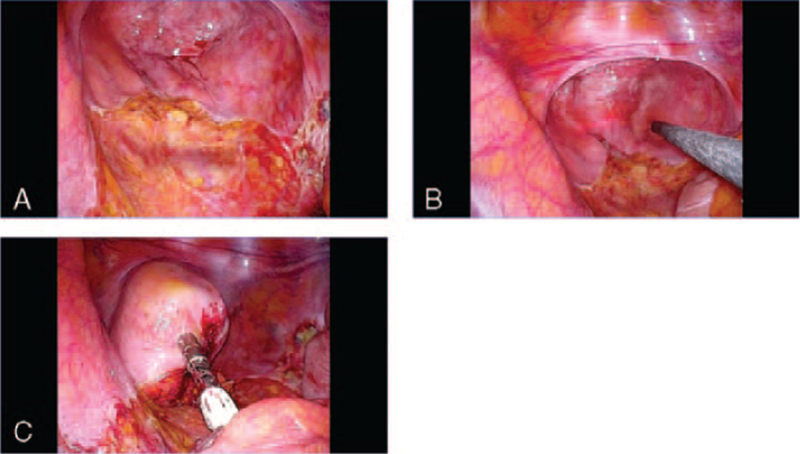
Representative intraoperative laparoscopic images at the reversal of Hartmann procedure. (A) The intraoperative image shows no postoperative adhesions in the pelvic space or around the rectal stump. (B) An intraoperative endoscopic leak test is performed and (C) end-to-end anastomosis with the stump of the rectum is performed according to the double stapling technique.

The abdominal cavity and subsequent laparoscopic surgery were performed via SILS from the stoma site (Fig. 2C, D). No postoperative inflammatory adhesions were observed in the pelvis or around the rectal end (Fig. 3A). After an intraoperative endoscopic leak test was performed (Fig. 3B), end-to-end anastomosis with the stump of the rectum was performed using the double stapling technique without adhesion detachment (Fig. 3C). The operative time for the gastrointestinal part of the procedure was 80 minutes, with minimal blood loss.

After RHP, the patient was placed in the supine position. The liver team performed a laparoscopic partial resection of the metastatic liver tumor. The patient's postoperative course was uncomplicated, and he was discharged from the hospital on postoperative day 7. Approximately 3 months later, when the transverse colostomy was closed, the patient was still in remission.

## 3 Discussion

Left-sided colorectal cancer with symptoms of intestinal obstruction often requires emergency surgery. The risk of anastomotic leakage and other postoperative complications is relatively common in emergency surgery. For patients who do not wish to have a permanent stoma, 1 common preventive tactic is to create a diverting stoma at the end of the ileum or at the mid-transverse colon, which makes stoma closure relatively easy and safe. However, although the addition of a diverting stoma can prevent serious complications, it does not reduce the incidence of anastomotic leakage.^[6]^ In cases where pretreatment is not possible and there is severe fecal retention in the abdominal cavity, there is a risk of severe intra-abdominal abscess or generalized peritonitis resulting from anastomotic leakage. In the presence of severe fecal retention, HP is the safest approach because there is no anastomosis. However, the technical difficulty and potential for postoperative complications associated with RHP make it a poor choice for surgeons when considering a temporary colostomy. According to a previous study, the main reason for the technical difficulty is the high degree of adhesions in the pelvis around the rectal stump.^[7]^ Generally, SILS is a more advanced minimally invasive surgical technique but has some technical drawbacks such as limited inline viewing and insufficient traction, which can be stressful for the surgeon,^[8]^ In the present case, the inflammatory adhesion control was good; thus, SILS from the stoma site could be performed from intraperitoneal observation to anastomosis without stress. If appropriate prevention of adhesions from laparoscopic HP is possible and laparoscopic RHP becomes easier, safe HP may become a first-line surgical technique for all patients with severe fecal retention.

Postoperative adhesions due to surgical procedures are the result of damage to the peritoneum and tissue, which causes the formation of fibrin.^[9]^ Thus, reducing tissue damage through good surgical manipulation is a major goal. Currently, various drugs are used as adhesion barriers in addition to surgical procedures. AdSpray is a safe and versatile drug approved for insurance cover in Japan that is in the form of a bioresorbable hydrogel consisting of N-hydroxysuccinimide-modified carboxymethyl dextrin, which can be used as a spray using the kit provided. The tip of the kit is cylindrical with a diameter of 3 mm and can be easily inserted into the abdominal cavity through a 5 mm port, making it easy to use in laparoscopic surgery. Because it is a spray-type drug, it can be applied evenly to targets with three-dimensional structures, such as the pelvis. It has been reported to be effective in preventing adhesions in large animal models^[10]^ and in clinical reports of hepatectomy.^[5]^As there has been no standard scale to evaluate the severity of adhesion, it was difficult to give a precise expression in this case. Using the TORAD score,^[11]^ which was reported to enable accurate evaluation of the severity of adhesion at repeat hepatectomy, the (A) score was 1 point because the pelvic surgery could be performed without any problem with SILS, and the (B) score was 1 point because dissection of adhesions was not necessary, and the overall score was considered EASY in this case. Thus, the application of AdSpray onto the pelvis, including the rectal stump, may be useful in patients with HP who do not wish to have a permanent stoma.

In this case, we decided to perform RHP on postoperative day 30, which is earlier than previously reported,^[12,13]^ to simultaneously perform liver surgery for synchronous liver metastasis of colorectal cancer, which was observed during the HP. It is a controversial issue whether to perform simultaneous surgery or nonsynchronous resection for the treatment of simultaneous liver metastases, but in our department, we performed a planned 2-stage resection for patient safety and adequate liver examination.^[14]^ As for the timing of the RHP, a longer interval (>9 months) from HP to RHP is associated with a higher risk of postoperative complications^[15]^ and a decline in the quality of life.^[16,17]^ Specifically, the treatment of highly advanced cancer with obstructive enterocolitis, such as liver metastasis, often requires chemotherapy or 2-stage surgery, as in this case, which places a heavy psychological and physical burden on the patient. Therefore, we considered early reversal as a reasonable option in this case to improve the quality of life and safety. Given that we only reported our experience with a single case, we plan to accumulate more cases in the future to confirm our findings.

In conclusion, Adspray was easy to use for three-dimensional fields such as the pelvis and was effective in preventing postoperative inflammatory adhesions during laparoscopic HP. Thus, RHP may become less risky and may be performed more as a minimally invasive surgery.

### 3.1 Informed consent

Written informed consent was obtained from the patient for the publication of this report.

## Acknowledgments

We are grateful for the cooperation of the personnel from the liver team at the Department of General and Gastroenterological Surgery, Osaka Medical and Pharmaceutical University, who were involved in partial resection of the liver procedure. We also thank Editage (www.editage.jp) for the English language editing.

## Author contributions

WO, MY, SM, HH, MI, KI, KTani, TK, YS, KTana, and JO performed the operation and provided perioperative care. WO designed and drafted the manuscript. MY, SM, HH, MI, KI, KTani, TK, YS, KTana, JO, and KU analyzed and interpreted the data. MY reviewed and revised the manuscript. KU supervised the patient's treatment and writing of the report. All authors have approved the final manuscript.

**Conceptualization:** Wataru Osumi.

**Data curation:** Wataru Osumi, Shinsuke Masubuchi, Hiroki Hamamoto, Masatsugu Ishii, Keisuke Izuhara, Kohei Taniguchi, Toru Kuramoto, Yusuke Suzuki, Keitaro Tanaka, Junji Okuda.

**Investigation:** Wataru Osumi, Shinsuke Masubuchi, Hiroki Hamamoto, Masatsugu Ishii, Keisuke Izuhara, Kohei Taniguchi, Toru Kuramoto, Yusuke Suzuki, Keitaro Tanaka, Junji Okuda.

**Project administration:** Wataru Osumi, Masashi Yamamoto.

**Supervision:** Kazuhisa Uchiyama.

**Writing – original draft:** Wataru Osumi.

**Writing – review & editing:** Wataru Osumi.
